# Cell Wall-Degrading Enzymes Differentially Mediate Aleurone Cell Release and Structural Integrity in Wheat Bran

**DOI:** 10.3390/foods15213810

**Published:** 2026-10-24

**Authors:** Zhongwei Chen, Chunlei Yu, Tingting Li, Huijun Wang, Bin Xu

**Affiliations:** School of Food Science and Engineering, Jiangsu University, Zhenjiang 212013, China; xxgzyxzh@163.com (C.Y.); 17853445315@163.com (T.L.); hjwang_work@163.com (H.W.); food_oil@126.com (B.X.)

**Keywords:** wheat bran, aleurone layer, cell wall, xylanase, pectinase

## Abstract

The aleurone layer (AL) is the most nutrient-rich fraction of wheat bran. However, its tight adhesion to the outer pericarp (OP) and intermediate layer (IL) within wheat bran limits its application in foods. In this study, cell wall-degrading enzymes (xylanase, pectinase, and β-glucanase) were employed to release the AL and its cells from wheat bran and to elucidate the underlying mechanism of this process. The results showed that all cell wall-degrading enzymes exhibited limited capacity to degrade the OP and IL. Xylanase and pectinase were the most effective in inducing AL detachment from wheat bran and intercellular dissociation of AL cells, whereas β-glucanase exerted a negligible effect. In comparison, xylanase enhanced the release of cell wall polysaccharides and phenolic acids, with ferulic acid release from the AL reaching 8.91 mg/g after treatment, but it also caused partial structural damage to AL cells. In contrast, pectinase caused less disruption to the AL cell walls and therefore better preserved cell integrity. The β-glucanase hydrolyzed β-glucan in the AL cell wall into glucose (22.17 mg/g) but did not lead to cell dissociation, plausibly because arabinoxylans overlay β-glucans and restrict enzyme access. Using coarse wheat bran (20–40 mesh) as the starting material yielded a higher proportion of intact AL cell-rich fraction than using a fine bran fraction (80–100 mesh). These findings highlight pectinase-assisted hydrolysis as an effective strategy for releasing intact aleurone cells for food applications.

## 1. Introduction

Wheat bran (WB), the primary byproduct of wheat flour milling, is well-known for its health benefits in reducing the incidence of chronic diseases, including type 2 diabetes, cardiovascular diseases, and certain types of cancers [1,2]. Despite these well-known nutritional and health benefits, the current industrial utilization of WB remains largely confined to low-value applications such as animal feed and fermentation feedstock [3], with limited exploitation in high-value food manufacturing [4]. As a heterogeneous multilayered tissue, WB consists of three structurally and chemically distinct layers: the outer pericarp (OP), intermediate layer (IL, inner pericarp, testa and hyaline layer) and aleurone layer (AL) [5]. The nutritional and bioactive components in WB are unevenly distributed across these layers, and are enriched in the AL [1]. The OP and IL are enriched in cellulose, lignin and pigments, which inevitably affect the color, texture, and sensory quality of WB-enriched foods [6,7,8]. OP and IL account for 27% and 26% of wheat bran, respectively [9]. They are preferential sites for the accumulation of microbial contaminants and several undesirable compounds, including pesticide residues and mycotoxins. Previous studies have demonstrated that removing the OP and IL by debranning can effectively reduce microbial contamination and decrease the levels of certain pesticide residues and mycotoxins in wheat kernels [10,11,12]. The AL serves as the dominant storage site for the nutritional and bioactive components of WB, where most of its soluble dietary fiber, minerals, proteins, ferulic acid, and B vitamins are concentrated [1,13]. The AL also offers additional advantages for food applications, including a lighter color, better digestibility, and lower contaminant loads relative to the outer bran layers [14,15]. Therefore, isolating the AL from other bran layers (i.e., OP and IL) represents a critical strategy for enhancing the food application potential of WB.

The separation of the AL from WB is predominantly achieved via two technical routes: dry fractionation and wet fractionation. Dry fractionation relies on an initial grinding step to break the physical adhesion between the AL and surrounding bran layers, with subsequent purification performed using air classification, sieving, or electrostatic separation [16,17]. This approach enables the production of AL-rich fractions with purities ranging from 60% to 90% [15], and offers key benefits, including the retention of water-soluble bioactive compounds and relatively low energy consumption. However, this method is limited by the low recovery yield of high-purity AL products and the strict requirements for equipment. Wet fractionation using chemical reagents is also commonly employed to extract WB components [16,18]; however, this method requires the use of large amounts of chemical reagents, which not only poses severe environmental concerns, but also leads to chemical residues in the final product [16].

In comparison, enzymatic treatment has the advantages of mild reaction conditions, high substrate specificity, no chemical residue, and environmental friendliness, and has therefore become a research hotspot for the efficient separation of cereal bran components. Cell wall-degrading enzymes, including cellulase, xylanase, and β-glucanase, can hydrolyze WB cell walls and weaken the cohesion between the AL and other bran layers, thereby facilitating the isolation of the AL [19]. Because the cell walls of the AL mainly consist of arabinoxylans (AXs, more than 60%) and β-glucans (~28%) [20], xylanase-based enzymatic treatment inevitably compromises the structural integrity of AL cell walls. Previous studies have demonstrated that cellulase (‘Onozuka’ R-10), Micelase (rich in cellulase and glucanase), and β-1,4-endoxylanase can weaken the integrity of AL cell walls [21], thus enabling the preparation of aleurone protoplasts from barley [22] and oat [23]. Aleurone protoplasts are mostly used for plant cell biology research but cannot meet the stability requirements of food processing and storage. An alternative strategy is to selectively weaken the intercellular adhesion between adjacent AL cells without impairing the native structural integrity of their cell walls. In plant tissues, adjacent cells and different tissues are connected by the middle lamella, a thin layer rich in pectin that serves as the intercellular adhesive material [24]. Notably, the middle lamella of wheat AL cells contains a trace amount of pectin [25,26]. Therefore, pectinase may have a relatively limited effect on the structural polysaccharides of aleurone cell walls while potentially facilitating cell separation by affecting pectin-containing intercellular adhesion regions. However, direct evidence demonstrating the selective degradation of the middle lamella by pectinase is still lacking. Pectinase may facilitate AL cell separation through pectin degradation, with possible contributions from accessory activities. Intact aleurone cells with preserved cell walls have better processing stability and nutrient retention capacity, and are therefore more suitable for the development of functional foods. However, no study has yet reported the enzymatic preparation of intact aleurone cells with preserved cell walls from WB.

This study aimed to isolate intact AL cells from WB using different enzymes, including cellulase, xylanase, β-glucanase, and pectinase. These enzymes were used to specifically target the hydrolysis of cell wall components and the disruption of intercellular adhesion in the OP, IL and AL to dissociate the AL while preserving the integrity of AL cell walls. Furthermore, the microstructures and chemical compositions of the isolated AL cells were investigated. The findings of this study will fill the technical gap in the enzymatic preparation of intact aleurone cells from WB and provide critical technical and theoretical support for the high-value food application of WB.

## 2. Materials and Methods

### 2.1. Materials and Chemicals

Wheat kernels (Jimai 22) were purchased from the Shandong Academy of Agricultural Sciences. WB was obtained from Chen Ke Ming Food Manufacturing Co., Ltd. (Suiping, Henan, China). Cellulase “Onozuka R-10” (>1000 U/g, including cellulase, pectinase and hemicellulase) was purchased from Yakult Honsha Co., Ltd. (Tokyo, Japan). Neutral protease (50 U/g), β-glucanase (>150 U/mg), xylanase (6000 U/mg), and pectinase (500 U/mg from *Aspergillus Niger*) with high purity (>98%) were purchased from the Sinopharm Group (Beijing, China). The chemicals 3,4,5-trimethoxy cinnamic acid, methanol, ethyl acetate, trans-ferulic acid, *p*-coumaric acid, trifluoroacetic acid, acetonitrile, ammonium molybdate, sodium sulfite and catechol were purchased from Yien Chemical Technology Co., Ltd. (Shanghai, China). Other chemical reagents used in the experiments were purchased from the Sinopharm Group (Beijing, China).

### 2.2. Preparation of Wheat Bran Layers and Wheat Bran Fractions

The AL, IL, and OP were obtained according to the method of Fulcher et al. [27]. Wheat kernels were soaked in distilled water at 4 °C for 24 h. First, the OP was peeled using tweezers, after which the germ and starchy endosperm were removed with a scalpel. Then, the AL was scraped away from the IL with the aid of a stereomicroscope. The dissected WB layers were air-dried and finally stored in a desiccator before analysis.

WB fractions with different particle sizes were obtained by centrifugal impact milling (CIM) and sieving [28]. During CIM, the bran fractions were impacted and sheared by the high-speed rotating rotor-tooth (6000 rpm) and the gear ring in the crushing chamber. CIM promoted the detachment of wheat bran layers. The AL in the bran fraction passing through 200 μm (~80 mesh) was completely detached in a single layer-cell cluster with 20–30 cells [28]. Therefore, after CIM, the WB fraction that passed through an 80-mesh sieve but was retained on the 120-mesh sieve was sealed in a desiccator and stored at 4 °C for further investigation.

### 2.3. Enzymatic Hydrolysis of Cell Walls of Wheat Bran

The WB fractions obtained by CIM and sieving were deproteinized using neutral protease (1200 U/g WB) to remove the residue protein from starchy endosperm that may interfere with subsequent enzymatic hydrolysis of the cell walls. After that, WB was destarched using α-amylase (4000 U/g WB) and glucoamylase (1000 U/g WB) at 50 °C until no blue color was observed upon iodine staining. Then, the WB fractions were suspended in 0.1 mol/L sodium acetate buffer (pH 5.0) at a ratio of 1:20 (*w*/*v*). Enzymatic hydrolysis was conducted using cellulase (“Onozuka” R-10) (>100 U/g WB), xylanase (240,000 U/g WB), β-glucanase (>120,000 U/g WB), or pectinase (200,000 U/g WB) at 35 °C in a shaking incubator at 150 rpm for 8 h, separately. Based on the method above, xylanase, β-glucanase, and pectinase were also applied to the AL, IL, and OP for 8 h, respectively. The concentrations of individual enzymes were selected based on preliminary experiments and the chemical composition of WB cell walls and the substrate specificity of each enzyme. The relatively higher concentration of cellulase and pectinase was used because their relatively low specific activity. Cellulose and pectic polysaccharides are structurally embedded within the WB cell wall matrix and require sufficient enzymatic activity for effective disruption, whereas xylanase and β-glucanase were applied at lower concentrations due to their higher catalytic efficiency toward their corresponding substrates [29].

Following enzymatic treatments, the residues and hydrolysates were separated by centrifugation (315K, Xi Ma centrifuge Co., Ltd., Yangzhou, China) at 6000 rpm for 10 min. The residues were washed three times with distilled water, and the supernatants were combined to ensure complete recovery of soluble components. The soluble fraction was concentrated by rotary evaporation (90 °C) and freeze-dried (Pilot 10-15S, Biocool experimental instrument Co., Ltd., Beijing, China) under vacuum for 48 h before weighing. The insoluble fraction was mixed with distilled water and stirred at 600 rpm for 2 h using an electric stirrer (JB90-S, Meiyingpu Instrument Manufacturing Co., Ltd., Shanghai, China). The insoluble fractions were filtered with 200-, 400- and 450-mesh sieves to obtain three fractions with different particle sizes, and were then freeze-dried for 48 h.

### 2.4. Chemical Component Analysis

The moisture, protein and ash content in WB samples were determined according to AACC 44-15, 46-10 and 08-01, respectively [30]. The total phosphorus content was determined using the AOAC official method 965.17 [31].

The content of phenolic acid was analyzed according to the method of Antoine et al. [32]. A 20.0 mg sample was hydrolyzed under nitrogen at 35 °C for 2 h in the dark with 2.0 mol/L NaOH (10.0 mL). Thereafter, the internal standard, 3,4,5-tri-methoxy-cinnamic acid, was added to the sample, and the mixture was adjusted to pH 2.0 with 6.0 mol/L HCl. Phenolic acid was extracted twice with three volumes of ethyl acetate. The ethyl acetate phases were evaporated and concentrated to 2–3 mL at 35 °C, then dried with nitrogen and stored in the dark. The residue was dissolved in 1.0 mL methanol (50% *v*/*v*), filtered (0.45 μm) and injected (20.0 μL) into an Eclipse Plus Phenyl-Hexyl column (4.6 × 250 mm, ZORBAX, Agilent, Santa Clara, CA, USA). Then samples were analyzed by HPLC with a UV detector set at 325 nm (Agilent Technology Co., Ltd., Santa Clara, CA, USA). The mobile phase consisted of solvent A (1.0 mmol/L TFA) and solvent B (acetonitrile/TFA, 90: 10, *v*/*v*). Linear gradient elution was carried out at 1.0 mL/min at 45 °C. The gradient shifted from 85/15 to 82/18 in 15 min, from 82/18 to 80/20 in 5 min, from 80/20 to 70/30 in 5 min, from 70/30 to 60/40 in 30 min, and from 60/40 to 85/15 in 3 min, and was then maintained at 85/15 for 2 min. For phenolic acids in liquid samples, 4 mol/L NaOH (1.5 mL) was added to the enzymatic hydrolysate (0.5 mL) and reacted at room temperature in the dark for 1 h. The subsequent procedures were identical to those described for solid samples.

Polysaccharide composition was analyzed according to the method of Antonie et al. [26]. The enzymatic hydrolysate was concentrated at 90 °C to 2 mL and centrifuged at 14,000 rpm for 5 min. The sediment was washed twice with distilled water. The combined supernatants were mixed with three volumes of ethyl alcohol and precipitated at 4 °C for 12 h. The mixture was centrifuged at 6000 rpm for 5 min. The sediment was washed twice with 75% ethanol and dried in an oven at 50 °C to obtain the polysaccharide fraction. Two mol/L TFA (4.0 mL) was added to the sample (10 mg) and hydrolyzed at 110 °C for 8 h. The hydrolysate was then concentrated to dryness at 80 °C. To remove residual TFA, 1 mL methanol was added and concentrated until dry. Subsequently, hydroxylamine hydrochloride (20 mg), allose (1 mg), and pyridine (1 mL) were allowed to react at 90 °C for 30 min. Then, acetic anhydride (1 mL) was added and reacted at 90 °C for 30 min. After cooling, the distilled water (5 mL) was added to terminate the reaction, followed by extraction with dichloromethane (1 mL). The organic phase was filtered (0.22 μm) and injected (2.0 μL, a split ratio of 30:1) into a fused-silica capillary column (30 m × 0.25 mm × 0.25 μm, DB-5MS, Agilent, Santa Clara, CA, USA). Then, samples were analyzed by a gas chromatograph (Agilent 7890A, Agilent Technologies, Santa Clara, CA, USA) with FID. The detector temperature was set at 300 °C, and the injector temperature was 250 °C. The experiment was carried out at 40 mL/min under high-purity hydrogen. The initial temperature of the chromatographic column was maintained at 110 °C for 2 min, increased to 190 °C at 10 °C/min and maintained for 4 min, then increased to 230 °C at 4 °C/min and maintained for 8 min.

### 2.5. Light Microscopy

The samples were observed using an optical microscope (BX53, Olympus Co., Tokyo, Japan) equipped with a digital camera system (DP50, Olympus, Tokyo, Japan) under normal light and polarized light with a 530 nm first-order waveplate. Representative images were selected.

### 2.6. Scanning Electron Microscopy

The microstructure of the isolated aleurone cells was observed under a scanning electron microscope (Quanta 250 FEI, Hillsboro, OR, USA). A small amount of dried sample was mounted onto conductive copper tape and sputter-coated with platinum prior to imaging. Microscopic observation was performed at an accelerating voltage of 5.0 kV, with magnifications ranging from 800× to 2000×.

### 2.7. Confocal Laser Scanning Microscopy

Samples were double-stained with 0.1% (*w*/*v*) acid fuchsin (dissolved in 1.0% acetic acid aqueous solution) and 0.01% (*w*/*v*) fluorescent brightener 28 aqueous solution. The stained samples were observed under a confocal laser scanning microscope (Leica TCS SP5, Wetzlar, Germany). The excitation wavelengths were set at 405 nm for the fluorescent brightener and 540 nm for acid fuchsin, with emission wavelengths of 415 nm and 630 nm, respectively. Z-stack scanning was performed in dual-channel mode, and representative images were selected as the results.

### 2.8. Raman Spectroscopy

A DXR™ Raman microscope (Thermo Scientific™, Waltham, MA, USA) was used for all imaging experiments. Each sample (5.0 mg) was evenly dispersed onto a microscope slide and covered by a coverslip. The cell wall regions of the samples were identified under a light microscope and selected for Raman measurement. Raman spectra were acquired using a 532 nm laser with a spot size of 7 μm × 7 μm and a laser power of 10 mW over the wavenumber range of 400–2000 cm^−1^. Each sample was analyzed at three positions within a cell.

### 2.9. Particle Size Measurement

The particle size properties of the AL samples were determined by a Lalaser particle analyzer (Mastersizer 3000, Malvern, England). The wet method was employed using water as the medium at 25 °C (refraction index, 1.3; absorption, 0.1; particle shape, irregular). The particle size distribution in the range of 0.1–1000 μm and particle size distribution curves were obtained based on the volume percentage to characterize the particle sizes of the samples.

### 2.10. Statistical Analysis

All quantitative experiments, including enzymatic hydrolysis experiments with cellulase, xylanase, β-glucanase, and pectinase, were independently performed in triplicate (*n* = 3). The data are presented as mean ± standard deviation (SD). The HPLC data were processed using Agilent OpenLAB chromatography software 3.0, and GC data were analyzed using Agilent ChemStation software (Rev. B.03.01, Agilent Technologies, Santa Clara, CA, USA). Data from phenolic acids, polysaccharides, and Raman microspectroscopy were baseline-corrected and plotted using Origin 2021. Laser confocal microscopy images were processed using Imaris 9.0.1. Peak areas were calculated by Gaussian fitting. Statistical analyses were performed using SPSS 26.0. One-way analysis of variance (ANOVA) was used to evaluate significant differences among treatments, and differences were considered statistically significant at *p* < 0.05.

## 3. Results and Discussion

### 3.1. The Morphology and Microstructure of WB Layers

**Figure 1:**
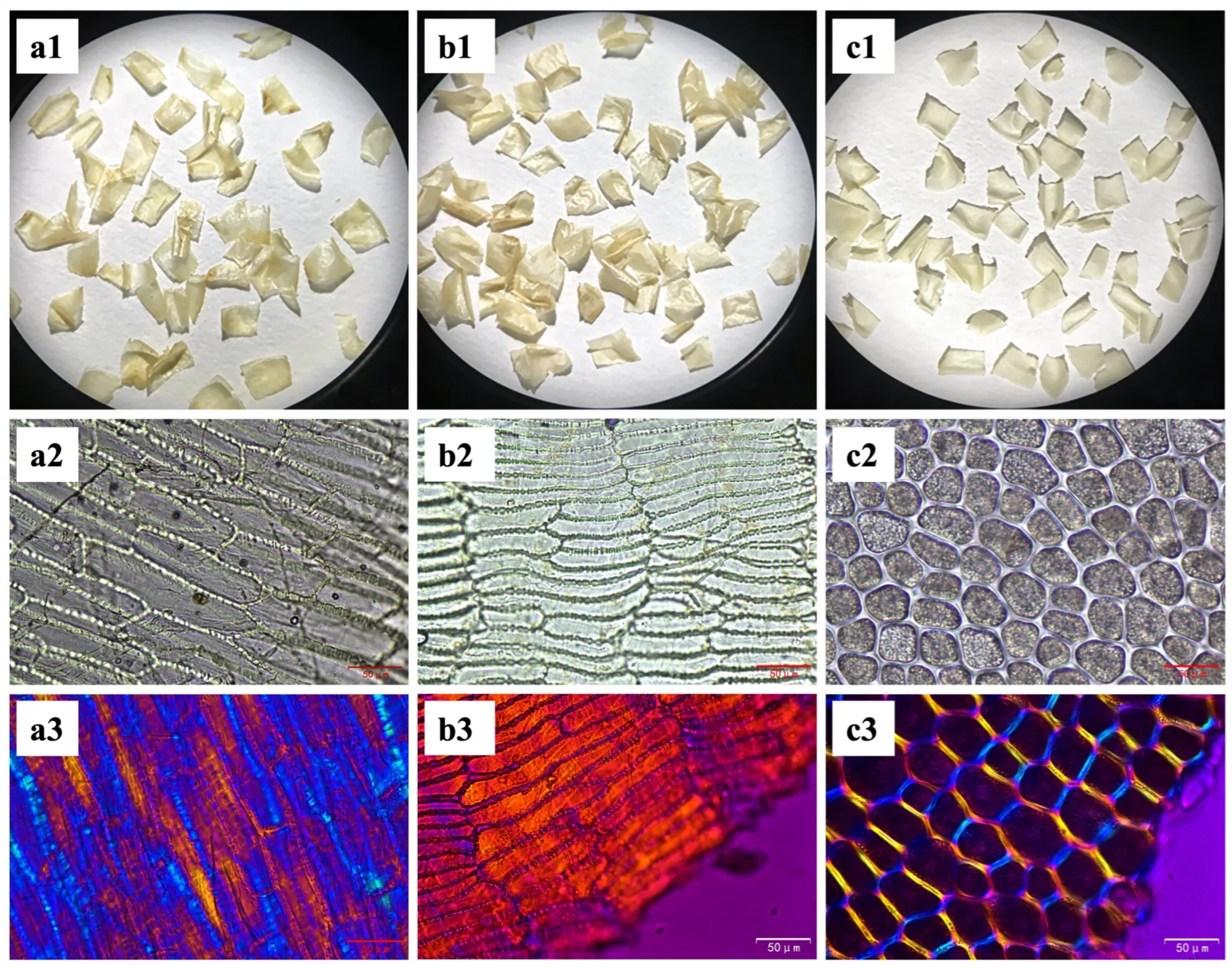
Micrographs of wheat bran layers obtained by manual isolation and observed by light microscopy. (**a1**–**c1**) The stereomicroscopic images of the outer pericarp (OP), intermediate layer (IL), and aleurone layer (AL), respectively; (**a2**–**c2**) the light micrographs of the OP, IL, and AL, respectively; (**a3**–**c3**) the polarized light microscopic images of the OP, IL, and AL, respectively.

Figure 1(a1–c1) shows the morphology of the WB layers (i.e., OP, IL and AL) obtained by manual peeling. The AL exhibited the lightest color among the three layers, while the IL appeared the most yellow due to the presence of pigments in the testa. The OP was light-yellow and partially translucent. Regarding the microstructure, the OP consisted of elongated cells with a spindle-like morphology, measuring 78 μm × 37 μm (*n* = 10, Figure 1(a2)). The IL was composed of transverse cells 21 μm in width and 146 μm in length (*n* = 10, Figure 1(b2)). The AL were composed of polygonal thick-walled cells with a diameter of 48 μm (*n* = 10, Figure 1(c2)) and exhibited lower light transmittance than the other layers under the microscope.

Under polarized light, the OP and IL exhibited a negative sign of birefringence (Figure 1(a3,b3)), whereas the AL had positive birefringence (Figure 1(c3)), which is in agreement with the results of the previous study [33]. The cellulose microfibers in the OP were relatively long (several hundred micrometers), whereas those in the IL were shorter (50–150 μm) and aligned parallel to each other. The AL cell walls displayed alternating orange and blue birefringence patterns, suggesting differences in polymer orientation within the cell wall. This birefringence phenomenon indicates the ordered arrangement of cell wall polymers. Similar observations were reported by Mense et al. [33], suggesting that birefringence can be employed to evaluate enzymatic hydrolysis efficiency.

### 3.2. Effects of Cell Wall-Degrading Enzymes on WB and Its Layers

**Figure 2:**
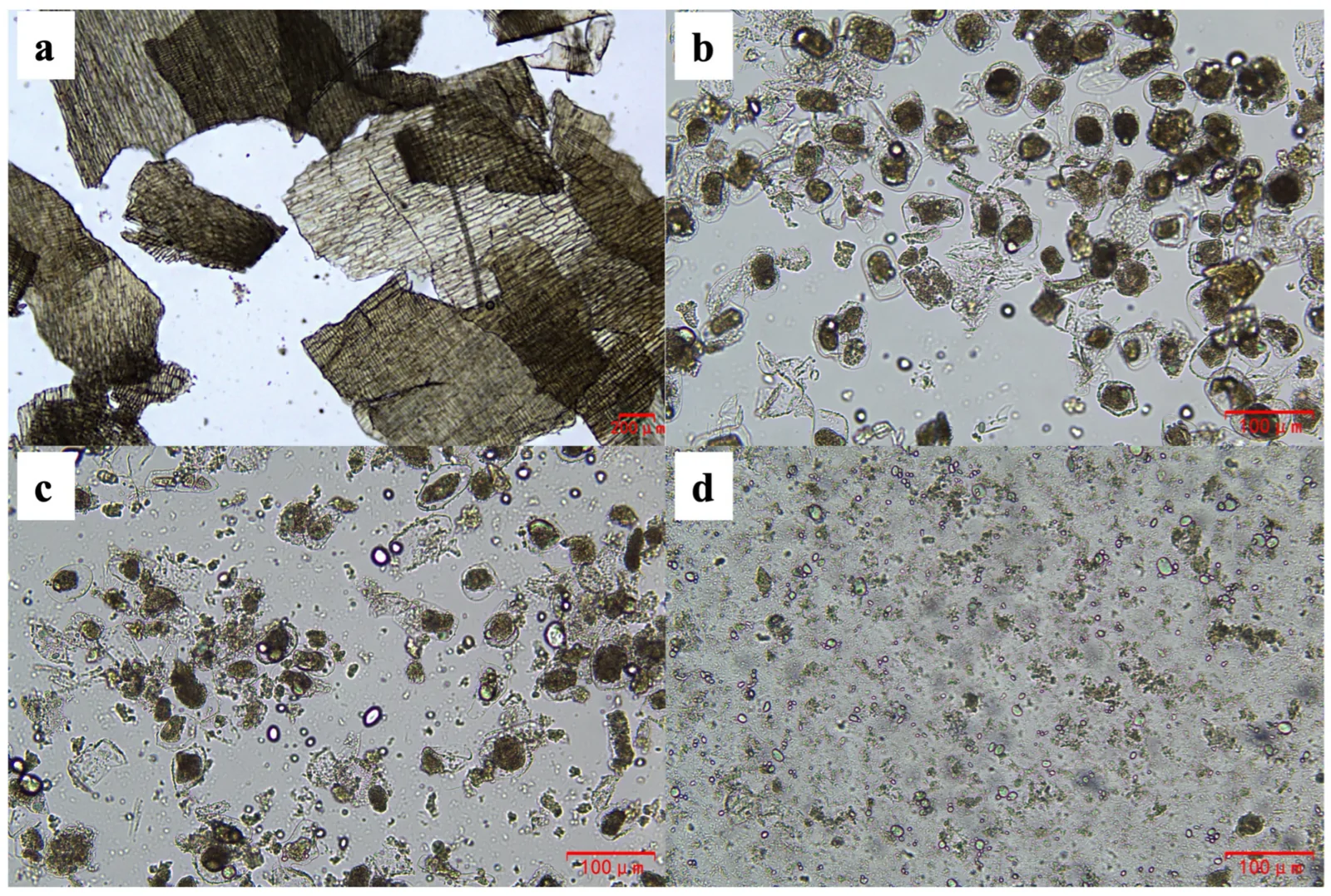
Micrographs of WB fractions after treatment with cellulase Onozuka R-10. (**a**) Fraction retained on a 200-mesh sieve; (**b**) fraction passing through a 200-mesh sieve but retained on a 400-mesh sieve; (**c**) fraction passing through a 400-mesh sieve but retained on a 450-mesh sieve; (**d**) fraction passing through a 450-mesh sieve.

Figure 2 presents microstructures of WB fractions after enzymatic treatments with “Onozuka R-10”. The fraction retained on the 200-mesh sieve mainly consisted of OP and IL. The fraction between 200- and 400-mesh was primarily composed of AL cells, whereas the 400–450-mesh fraction consisted mainly of cell contents mixed with a small amount of AL cells. No intact AL cells were observed in the fraction passing through the 450-mesh sieve. These results indicate that the mixed cell wall-degrading hydrolases could dissociate AL cells but had no significant effect on the cell walls of the OP and IL. This finding is consistent with previous studies by Hooley et al. [23] and Taiz et al. [34], in which cellulase (Onozuka R-10) was employed to isolate AL protoplasts from oat and barley, respectively.

The proportion of different bran fractions after treatment with Onozuka R-10 was investigated. The fraction over the 200-mesh sieve (F_(−200)_) accounted for the highest proportion (73.91%), followed by the fraction passing through the 450-mesh sieve (12.01%), while F_(200–400)_ and F_(400–450)_ accounted for 4.36% and 2.94%, respectively. No intact aleurone cells were detected in the F_(−200)_ fraction. The overall recovery of intact AL cells was low, and was attributed to partial or complete disruption of the AL cell wall by enzymatic hydrolysis, which caused leakage of intracellular contents. Therefore, this result indicates that, the Onozuka R-10, which contains compound cell wall-degrading enzymes disrupted the aleurone cell walls greatly, resulting in the release of intracellular components and a low recovery of intact aleurone cells.

**Figure 3:**
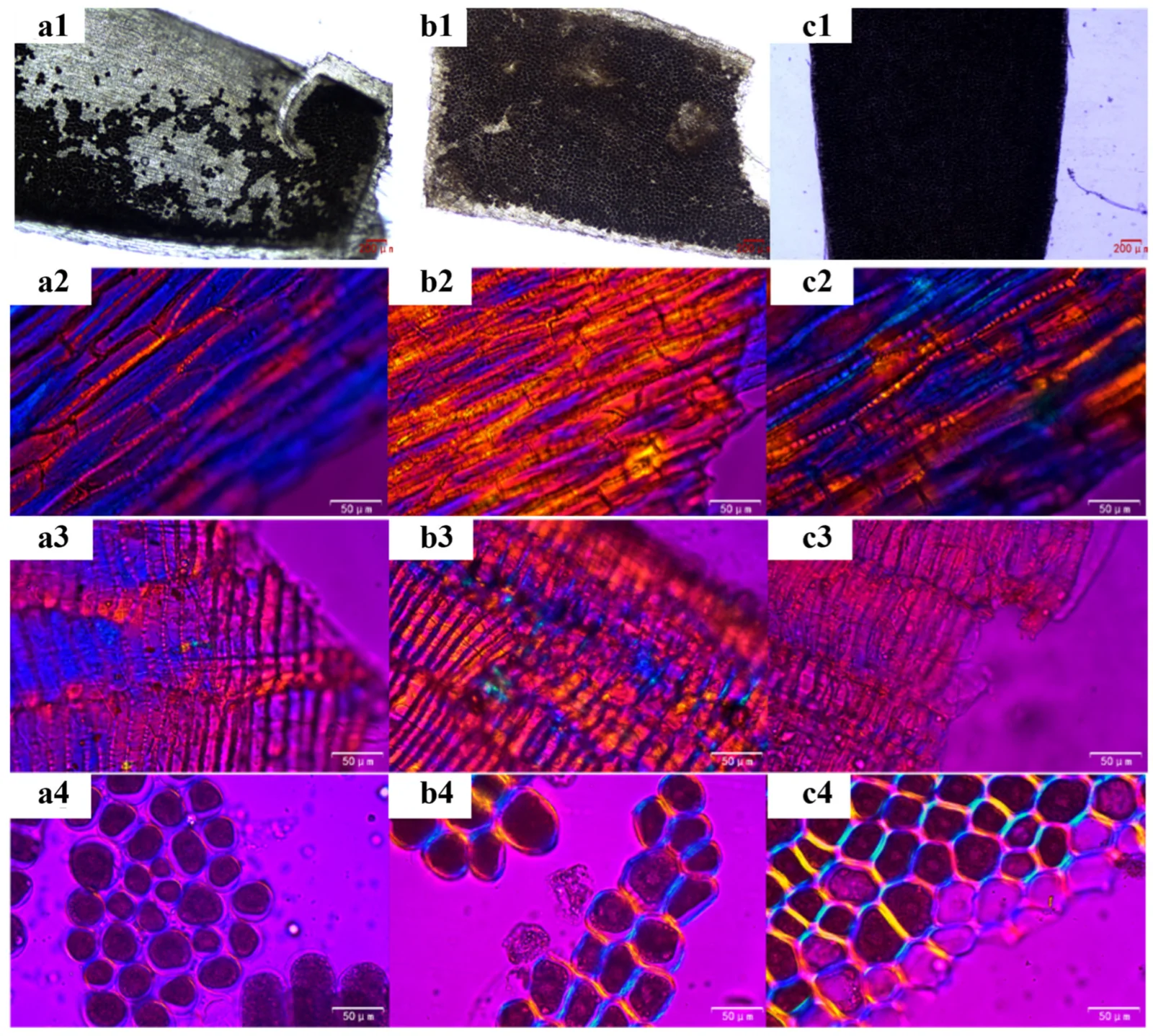
Micrographs of wheat bran and its dissociated layers after treatment with xylanase, pectinase, and β-glucanase. (**a1**–**c1**) WB after treatment with xylanase, pectinase, and β-glucanase, respectively; (**a2**–**c2**) microstructures of the outer pericarp after treatment with xylanase, pectinase, and β-glucanase, respectively; (**a3**–**c3**) polarized light micrographs of the IL after treatment with xylanase, pectinase, and β-glucanase, respectively; (**a4**–**c4**) polarized light micrographs of the AL after treatment with xylanase, pectinase, and β-glucanase, respectively.

Because the AL cell walls are mainly composed of arabinoxylans (~65%) and β-glucan (~28%), the effects of cell wall-degrading enzymes (xylanase, pectinase and β-glucanase) on WB and its layers were investigated. Figure 3 illustrates the effects of cell wall-degrading enzymes on WB and its layers. As shown in Figure 3(a1), approximately half of AL cells were dissociated from WB after xylanase treatment, whereas only a small number of AL cells were detached after pectinase treatment (Figure 3(b1)). β-glucanase treatment resulted in limited morphological changes in the AL under the observed conditions (Figure 3(c1)). These findings indicate that different enzymes exhibit distinct effects on the detachment of aleurone cells. The effects of xylanase, pectinase, and β-glucanase on WB layers were also examined. As shown in Figure 3(a2–c2), no obvious alterations in the microstructure or birefringence patterns of OP and IL were observed after treatment with xylanase, pectinase, or β-glucanase based on microscopic observation. This is attributed to the high content of cellulose in their cell walls, which is resistant to enzymatic hydrolysis. Moreover, AXs are tightly cross-linked to cellulose through ferulic ester linkages and lignin, limiting enzymatic degradation [35].

After xylanase treatment, the birefringence in the AL cell walls disappeared. In contrast, pectinase hydrolysis led to intercellular dissociation within the AL, while the birefringence of the AL cell wall was largely retained. This is likely because the AL cell wall contains minimal cellulose and lignin and exhibits a relatively low degree of cross-linking [21,36,37]. Compared with xylanase, pectinase exerted a weaker hydrolytic effect on AL cell walls and was more conducive to preserving cell integrity. Although β-glucanase released a considerable amount of glucose (22.17 mg/g), β-glucans are mainly located in the inner cell wall of the AL [38] and are not the principal intercellular cementing polysaccharides between AL cells. Instead, arabinoxylan-rich networks, together with ferulate-mediated crosslinks, appear to play a more dominant structural role. Therefore, hydrolysis of β-glucans alone was insufficient to disrupt cell–cell adhesion [39].

To explore the effect of enzyme treatment on the release of AL cells and its mechanism, the chemical composition of hydrolysates obtained after xylanase and β-glucanase treatments were analyzed for the WB and hand-isolated AL. As shown in Table 1, for the AL, xylanase treatment released a greater number of monosaccharides than β-glucanase treatment. The xylanase treatment exhibited a substantially greater monosaccharide-releasing capacity for the detached AL compared with WB, indicating that the AL cell wall was more susceptible to xylanase than the cell walls of OP and IL. In the hydrolysates of xylanase-treated AL, the ratio of arabinose to xylose (A/X) was 0.375, suggesting that the degraded AXs had a relatively low substitution degree. Previous studies have reported that moderately substituted AXs in the AL cell wall (A/X = 0.3–0.4) can be completely hydrolyzed at higher xylanase concentrations, whereas the cell walls of the OP and IL remain intact, as they contain highly substituted AXs and have a high lignin content [21]. The A/X ratio of the xylanase-treated WB hydrolysate was higher than 1.10, with arabinose (1.62 mg/g) slightly exceeding xylose (1.47 mg/g). Although the A/X ratio of the arabinoxylans in OP exceeded 1.10, the results indicate that the xylanase used in this study had some debranching or side activities, due to the existence of α-L-arabinofuranosidase [40].

The release of *p*-coumaric acid and ferulic acid, which are predominant phenolic acids in WB and its layers [41,42,43], after the enzymatic treatments was also investigated. The xylanase treatment released significantly higher amounts of both phenolic acids from isolated AL than from WB (*p* < 0.05). Specifically, hydrolysates of the AL contained 0.29 mg/g p-coumaric acid and 8.91 mg/g ferulic acid, which were 3.6-fold and 8.8-fold higher than the corresponding values from WB, respectively. The β-glucanase treatment showed no capacity to release phenolic acids from WB. Only a trace amount of ferulic acid (0.09 mg/g) was detected in the AL hydrolysate, and no phenolic acids were detected in WB hydrolysates after β-glucanase treatment. These results reveal that xylanase, rather than β-glucanase, is the primary enzyme responsible for degrading WB cell walls. Meanwhile, AL cell walls are more susceptible to enzymatic hydrolysis by xylanase than those of WB, whereas β-glucanase exerted negligible degradative effects on the WB cell walls.

### 3.3. Effects of Xylanase or Pectinase Treatments on AL Dissociation

**Figure 4:**
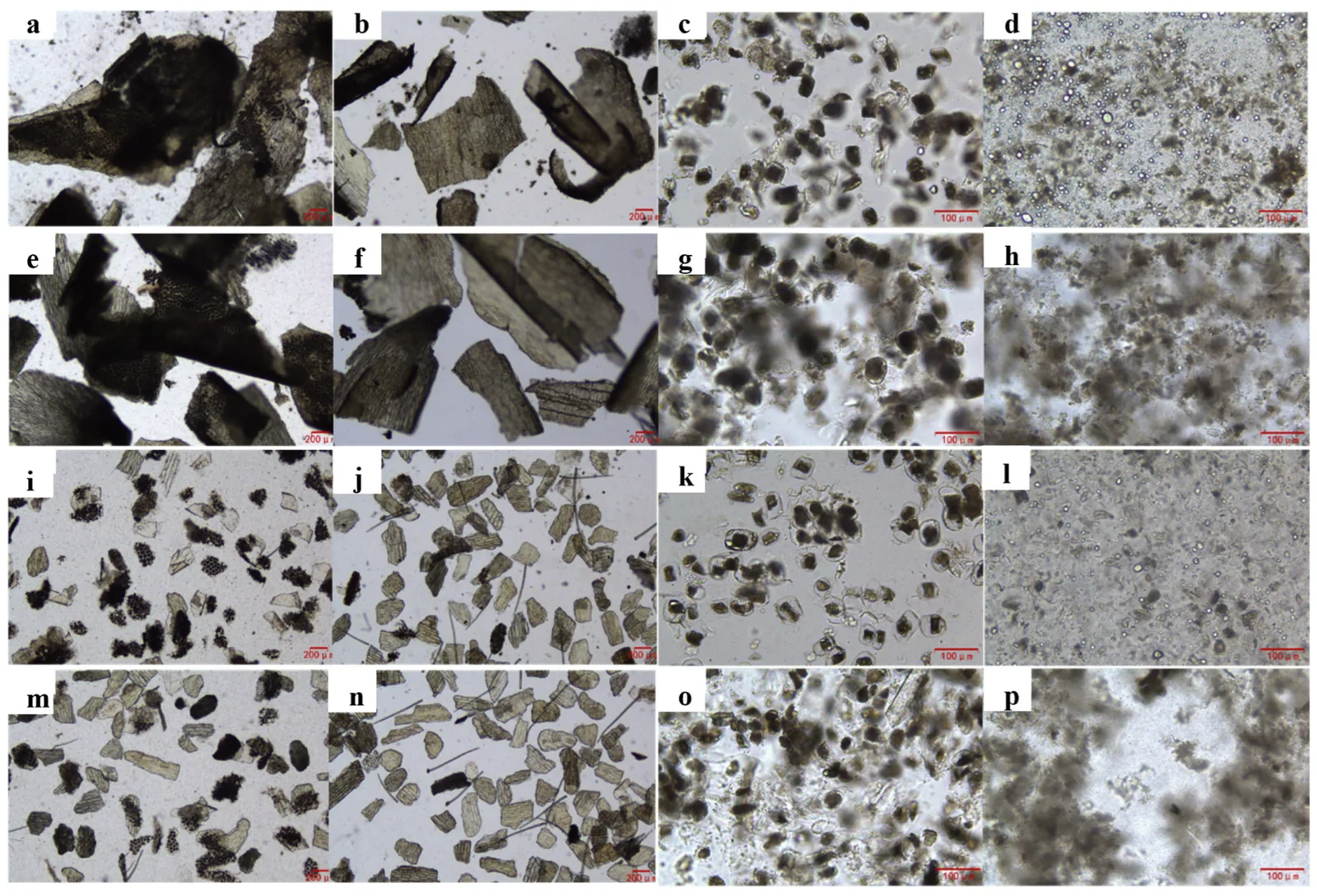
Light micrographs of WB and detached WB fractions after enzymatic treatments. (**a**–**d**) Micrographs of WB after xylanase treatment (before stirring and filtering), the fraction retained over the 200-mesh sieve (F_(−200)_), the fraction between the 200- and 450-mesh sieves (F_(200–450)_) and the fraction passing through the 450-mesh sieve (F_(450+)_), respectively. (**e**–**h**) Micrographs of WB_(20–40)_ after pectinase treatment, F_(−200)_, F_(200–450)_ and F_(450+)_, respectively. (**i**–**l**) Micrographs of WB_(80–100)_ after xylanase treatment, F_(−200)_, F_(200–450)_ and F_(450+)_, respectively. (**m**–**p**) Micrographs of WB_(80–100)_ after pectinase treatment, F_(−200)_, F_(200–450)_ and F_(450+)_, respectively.

As shown in Figure 4, after enzymatic treatment using xylanase or pectinase for 8 h, many aleurone cells were released from the WB (Figure 4a,b). After adequate stirring, most AL cells in the WB were separated from non-AL cells (Figure 4b,f) and enriched in the fraction between the 200- and 450-mesh sieves (F_(200–450)_). The OP and IL, which were more resistant to enzymatic degradation, were retained on the 200-mesh sieve (F_(−200)_), whereas AL cell contents and starch granules were enriched in the fraction passing through the 450-mesh sieve (F_(450+)_). For ground bran fraction (80–100 mesh), aleurone cells were still aggregated after enzymatic treatment (Figure 4i,m). After stirring, most aleurone cells in the detached bran fraction were separated and enriched in F_(200–450)_ (Figure 4k,o). For coarse WB, the non-AL particles (OP and IL) and the aleurone cell contents were enriched in F_(−200)_ and F_(450+)_, respectively.

As shown in Table 2, the amount of solubilized material released by xylanase treatment was substantially higher than that released by pectinase treatment, indicating that pectinase exerted a weaker degradative effect on AL cell walls. Pectinase treatment yielded a higher proportion of F_(200–450)_, which is rich in intact AL cells, than xylanase treatment. The proportion (weight basis) of the AL cell-rich fraction obtained from the detached WB fraction (WB_(80–100)_) was lower than that obtained from coarse bran, likely due to the reduced integrity of AL cells in fine bran particles. Xylanase hydrolysis yielded a higher proportion of 450-mesh passable material than pectinase treatment, likely due to more extensive disruption of AL cell walls, which increased susceptibility to fragmentation during mechanical stirring and promoted greater release of aleurone cell contents. Briefly, pectinase hydrolysis of coarse WB as the raw material achieved a higher yield (10–13%) of the AL cell-rich fraction, demonstrating the superiority of coarse WB over fine detached bran fractions for intact aleurone cell preparation.

The chemical compositions of the hydrolysates and residues obtained from the coarse bran (WB_(20–40)_) and fine bran fractions (WB_(80–100)_) with detached AL cell clusters after enzyme treatments were investigated. As shown in Table 3, F_(200–450)_ exhibited higher ash and phosphorus contents than F_(200+)_. The hydrolysate after xylanase treatment contained higher levels of phosphorus and phenolic acids, but lower protein content than that of pectinase hydrolysate. Since phosphorus in WB is mainly localized in the AL [44] and phenolic acids are cross-linked with AXs, these results indicate the higher release of AL cell contents and more extensive disruption of AL cell walls by xylanase. As to the residue fraction, the fraction of F_(−200_), mainly composed of the OP and IL, showed lower phosphorus, ash and protein contents, but relatively high levels of ferulic acid. Small amounts of protein (1.4–3.8%) were detected in F_(−200)_ because the IL and OP contain 1.7–4.5% protein [41]. Finer bran fractions (F_(200–450)_ and F_(450+)_) contained higher ash, phosphorus, phenolic acid, and protein contents than the coarser fraction (F_(−200)_), indicating the enrichment of aleurone cells and cell content. The soluble hydrolysates exhibited relatively high ash contents (~35%), which was mainly attributed to the enrichment of soluble mineral components released from WB during enzymatic disruption. Ash in WB is predominantly localized in the AL (14.4–17.2%) [1] and associated with phytate complexes. Enzymatic degradation of cell wall structures may facilitate the release of these mineral-containing components into the soluble fraction [9].

**Figure 5:**
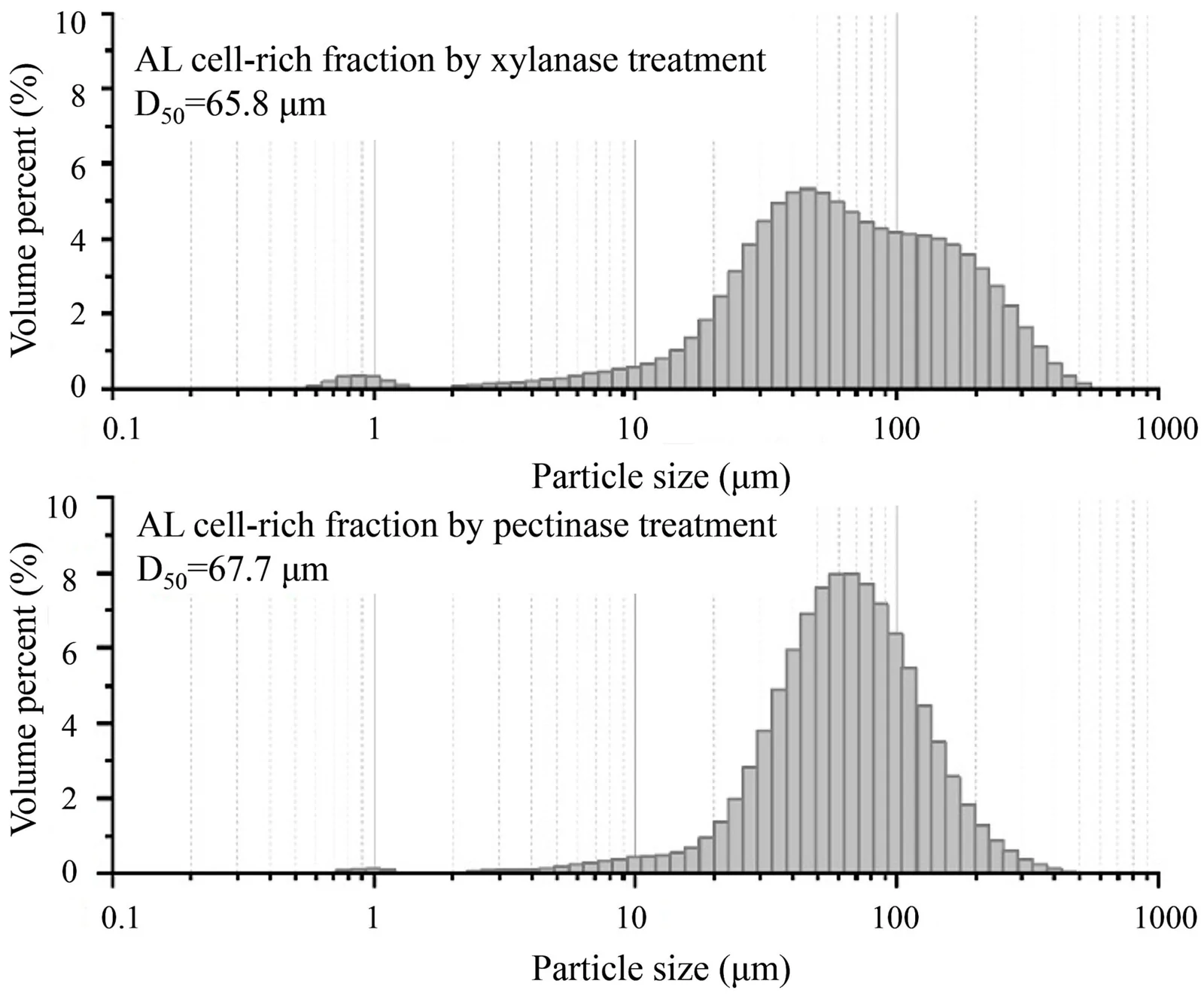
Particle size distributions of aleurone cell-rich fractions obtained from wheat bran by enzyme treatment with xylanase and pectinase.

**Figure 6:**
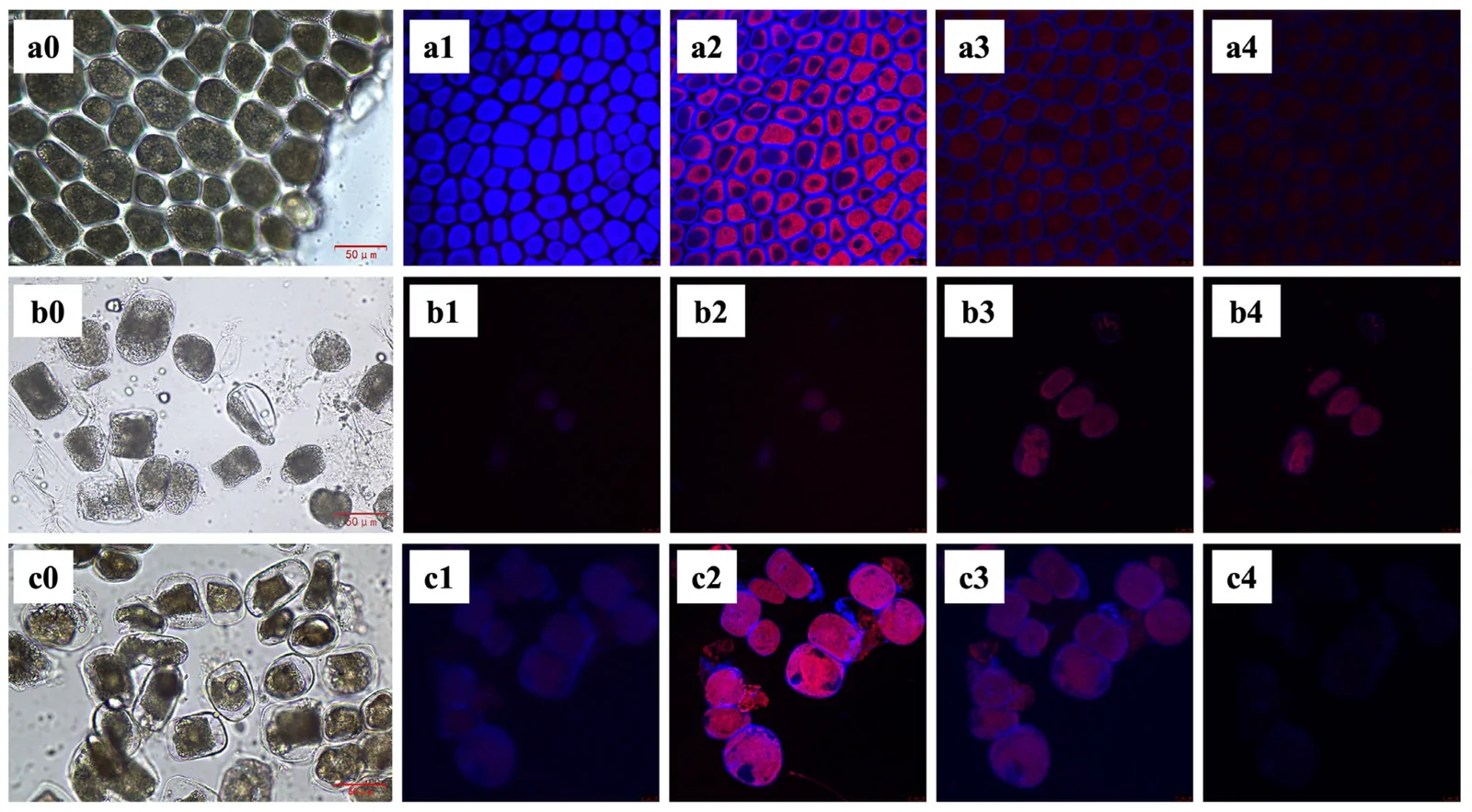
Micrographs of native AL and AL cells obtained by enzymatic treatments under light microscope and CLSM. (**a**) Native AL; (**b**) AL cells obtained by xylanase; (**c**) AL cells obtained by pectinase. (**a0**–**c0**) Optical micrographs. (**a1**–**a4**) Micrographs showing planar views of native AL at different depths; (**b1**–**b4**) micrographs showing planar views of AL cells obtained by xylanase at different depths; (**c1**–**c4**) micrographs showing planar views of AL cells obtained by pectinase at different depths.

**Figure 7:**
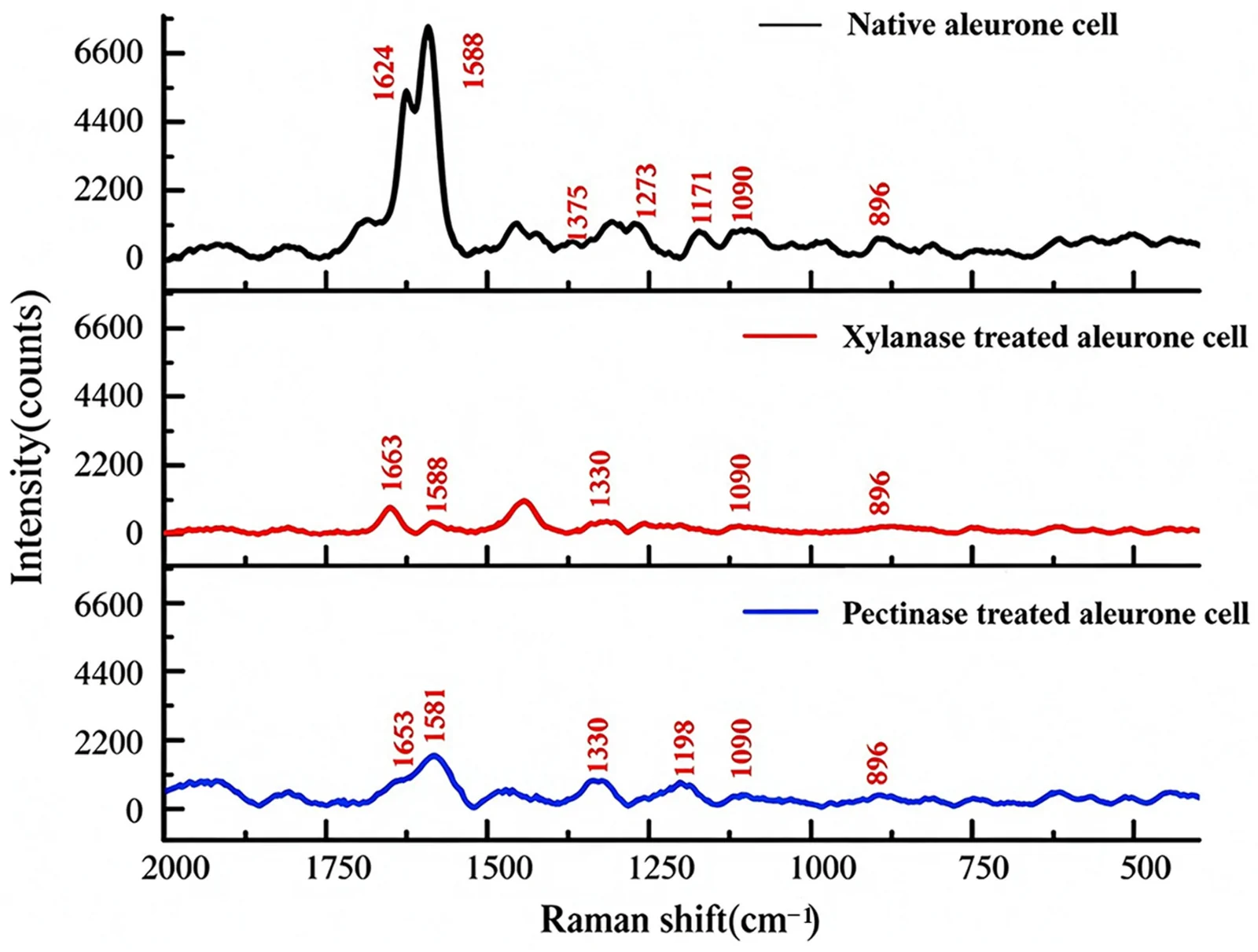
Laser Raman spectra of cell walls in the native AL and AL cells obtained by different enzymes.

To quantitatively evaluate the structural integrity of isolated AL cells, the particle size distributions of the AL cell-rich fractions were analyzed (Figure 5). The AL cell-rich fraction obtained by xylanase treatment exhibited a bimodal distribution, with peaks at 52 and 201 μm, whereas the AL cell-rich fraction obtained by pectinase treatment showed a relatively narrow unimodal distribution with a median particle size (D_50_) of 68 μm. The distinct particle size profiles reflect differences in swelling degree and purity between the two fractions. Arabinoxylans are major components of AL cell walls [45] and contribute significantly to cell wall rigidity. In contrast, pectin is only a minor constituent of the wall itself and occurs mainly in the middle lamella between adjacent AL cells. Therefore, pectinase treatment induces relatively limited cell wall degradation, resulting in a low degree of swelling. The particle size distribution results are consistent with microscopic observations (Figure 6) and Raman spectral analysis (Figure 7), supporting the higher purity and cell structural integrity obtained by pectinase treatment than that obtained by xylanase treatment.

### 3.4. Microstructure and Chemical Composition of Intact Aleurone Cells

Figure 6 showed the microstructures of native AL and the AL cells obtained after enzymatic treatments under a light microscope. After staining with fluorescent brightener and acid fuchsin, β-glucan and proteins in AL cells appeared blue and red, respectively. As shown in Figure 6(a1–a4), the cell wall of AL showed strong blue fluorescence uniformly, which is in agreement with a previous study by Wood et al. [38]. A concave structure was observed on the upper surface of the AL, corresponding to the side adjacent to the starchy endosperm. Unstained regions at the interfaces between adjacent cells indicated the presence of intercellular gaps. AL cells treated with pectinase exhibited stronger and more continuous blue fluorescence than those treated with xylanase (Figure 6b,c). The weaker fluorescence observed in xylanase-treated cells is likely associated with cell wall thinning (more compact, with an average thickness of 3.58 μm, *n* = 20) (Figure 6(b0)), which could reduce the total amount of accessible β-glucan for the calcofluor within the optical section, even though β-glucan is retained in the bulk residue. In comparison, the AL cell wall architecture with a cell wall thickness of 6.14 μm (*n* = 20) was better preserved after pectinase treatment (Figure 6(c0)).

Figure 7 shows the Raman spectra of AL cell walls. A weak peak at 899 cm^−1^, which corresponds to β-(1 → 4) glycosidic linkages, can be found in the spectra [46]. The spectral region of 1300–1400 cm^−1^ was associated with CH and COH bending vibrations, while the 1000–1200 cm^−1^ region corresponded to C-O-C, C-O, and C-C stretching vibrations, which are characteristic of AXs [47]. The peak values at 1628, 1599 and 1274 cm^−1^ are related to the stretching vibrations of the phenolic ring, which can be used to estimate the content of phenolic acids [48]. Compared to the Raman spectra of AL cell walls, it can be found that pronounced AX and ferulic acid peaks in the intact AL were stronger than those of AL cells obtained by xylanase and pectinase treatments, indicating partial degradation of AXs and release of phenolic acids during enzymatic hydrolysis. The ratio of phenolic acid to AXs reflects the degree of polysaccharide cross-linking of the AL cell wall, because ferulic acid can form dimers via biphenyl bridges, cross-linking adjacent AX chains and strengthening cell wall adhesion [48]. The Raman peak area ratios of phenolic acid to AX in the intact AL, xylanase-treated AL cells, and pectinase-treated AL cells were 2.27, 0.49, and 1.50, respectively. Accordingly, the mechanical strength of the cell walls in different samples followed the order Intact AL > pectinase-treated AL cells > xylanase-treated AL cells.

AL cells are rich in arabinoxylans, β-glucan, ferulic acid, and minerals. Therefore, the recovered intact AL cell fraction from WB could be considered as a promising functional ingredient for developing cereal foods. In the present study, β-glucanase was introduced to facilitate the disruption of cell wall architecture and promote the release of aleurone cells. The hydrolysis of β-glucan may generate lower-molecular-weight oligosaccharides, which may leach into the hydrolysate and reduce nutritional function of AL cells. From a techno-economic perspective, the present enzymatic process still requires further optimization before scale-up. First, the relatively long residence time (8 h hydrolysis at 35 °C) may reduce reactor productivity and increase operating costs at industrial scale. In addition, enzyme consumption is likely to constitute an important cost component, as the economic feasibility of lignocellulosic bioprocessing is often strongly affected by enzyme loading and enzyme price. Enzyme recovery and reuse may provide a potential route to reduce this cost. Immobilization, membrane-assisted recovery, adsorption-based recycling, or direct reuse of enzyme-containing liquid streams have all been investigated for lignocellulose-degrading enzymes [49]. Another limitation of the present process is the relatively low recovery of the fraction enriched in intact AL cells. Furthermore, although AL cells are theoretically expected to contain lower levels of pesticides and mycotoxins, this has not been experimentally verified in the present work. Future studies will systematically investigate the migration and accumulation patterns of typical food safety hazards during the AL cell isolation process. In addition, subsequent optimization efforts should aim to improve product recovery per unit mass of WB while lowering enzyme dosage and shortening hydrolysis time.

## 4. Conclusions

Different cell wall-degrading enzymes exhibited distinct effects on different WB layers (OP, IL, and AL) and AL cell dissociation. All three enzymes showed limited capacity to degrade the OP and IL. Xylanase and pectinase effectively degraded AL cell walls and promoted intercellular dissociation of AL cells, whereas β-glucanase exerted a negligible effect, probably due to the β-glucan located in the inner cell wall of AL. Xylanase exhibited stronger hydrolytic activity toward AL cell walls than pectinase, while pectinase treatment yielded AL cells with better-preserved cell walls and intracellular contents, making it superior for recovering structurally intact AL cells. This difference likely arises from the higher abundance of xylan relative to pectin in the AL cell wall matrix, where xylan serves as a more accessible and critical substrate for enzymatic hydrolysis. AL cells can be released from wheat bran through enzymatic treatment combined with mechanical stirring. The coarse bran fractions provided a higher recovery of the AL cell-enriched fraction than the fine bran fractions with detached AL cell clusters. Collectively, this work clarifies the differential degradation effects of cell wall-degrading enzymes on wheat bran layers, especially on the release of AL cells, providing a theoretical foundation for intact aleurone cell production and wheat bran valorization.

## Figures and Tables

**Table 1:** Monosaccharide and phenolic acid composition of enzymatic hydrolysates derived from wheat bran and the hand-isolated aleurone layer.

Starting Material	Enzymatic Treatment	Arabinose (mg/g)	Xylose (mg/g)	Glucose (mg/g)	Galactose (mg/g)	*p*-Coumaric Acid (mg/g)	Ferulic Acid (mg/g)
**Wheat bran**	Xylanase	1.62 ± 0.66 ^b^	1.47 ± 0.79 ^c^	0.34 ± 0.18 ^c^	0.18 ± 0.18 ^c^	0.08 ± 0.01 ^c^	1.01 ± 0.03 ^d^
	β-glucanase	0.90 ± 0.28 ^b^	0.53 ± 0.64 ^c^	0.35 ± 0.19 ^c^	0.35 ± 0.23 ^c^	nd	nd
**Aleurone layer**	Xylanase	19.19 ± 4.29 ^a^	51.15 ± 13.16 ^a^	32.33 ± 12.12 ^a^	2.04 ± 1.32 ^a^	0.29 ± 0.08 ^a^	8.91 ± 0.24 ^a^
	β-glucanase	1.47 ± 0.43 ^b^	0.82 ± 0.21 ^c^	22.17 ± 1.85 ^b^	nd	nd	0.09 ± 0.02 ^d^

Note: Different lowercase letters in the table indicate a significant difference between the same column of data (*p* < 0.05), and “nd” indicates no detection. The galactose value shown corresponds to neutral galactose only, owing to the limitation that conventional gas chromatography cannot detect uronic acids.

**Table 2:** Proportion of recovered soluble and residue fractions after enzymatic treatment and filtration of wheat bran (wet basis).

Starting Material	Enzyme	Soluble Substance (%)	F_(−200)_ (%)	F_(200–450)_ (%)	F_(450+)_ (%)
WB_(20–40)_	Xylanase	37.41 ± 3.63 ^ab^	59.84 ± 2.49 ^b^	8.91 ± 0.30 ^bc^	3.71 ± 0.20 ^b^
	Pectinase	22.51 ± 0.21 ^c^	60.96 ± 1.27 ^ab^	11.19 ± 0.30 ^ab^	2.71 ± 0.00 ^b^
WB_(80–00)_	Xylanase	37.52 ± 0.05 ^ab^	61.45 ± 0.27 ^ab^	7.88 ± 0.41 ^c^	2.43 ± 0.00 ^b^
	Pectinase	19.75 ± 2.62 ^c^	63.22 ± 0.02 ^a^	9.52 ± 0.51 ^bc^	1.93 ± 0.10 ^b^
Commercial WB	Xylanase	41.09 ± 0.37 ^a^	39.18 ± 1.09 ^c^	10.91 ± 1.93 ^ab^	8.87 ± 0.32 ^a^
	Pectinase	34.18 ± 0.37 ^b^	37.23 ± 1.07 ^c^	13.30 ± 1.45 ^a^	7.96 ± 2.25 ^a^

Note: WB_(20–40)_: coarse wheat bran fraction; WB_(80–100)_: the WB fraction with detached AL cell clusters after centrifugal impact milling; F_(−200)_: the fraction retained over the 200-mesh sieve; F_(200–450)_: the fraction retained between the 200- and 450-mesh sieves; F_(450+)_: the fraction passing through the 450-mesh sieve; the proportions of soluble and residue fractions were calculated on a wet basis. The total mass may exceed 100% owing to the high hygroscopicity of saccharides, which hinders thorough sample drying; different superscript letters in the table indicate a significant difference between the same column of data (*p* < 0.05).

**Table 3:** Chemical composition of soluble hydrolysates and residue fractions obtained from wheat bran after enzymatic treatment (dry basis).

Sample	Starting Material	Enzyme Treatment	Ash (%)	Phosphorus (mg/g)	*p*-Coumaric Acid (mg/g)	Trans-Ferulic Acid (mg/g)	Protein (%)
Hydrolysate	WB_(20–40)_	Xylanase	35.94 ± 0.75	27.58 ± 2.06 ^a^	0.02 ± 0.01	4.58 ± 0.71 ^a^	14.04 ± 1.30 ^b^
		Pectinase	36.32 ± 3.37	17.39 ± 4.04 ^b^	nd	0.38 ± 0.00 ^c^	17.58 ± 0.92 ^a^
	WB_(80–100)_	Xylanase	36.32 ± 1.86	25.61 ± 0.19 ^a^	0.10 ± 0.00	2.05 ± 0.00 ^b^	12.13 ± 0.06 ^b^
		Pectinase	34.98 ± 1.08	16.11 ± 0.42 ^b^	nd	0.56 ± 0.00 ^c^	13.20 ± 0.77 ^b^
Residue fraction of F_(−200)_	WB_(20–40)_	Xylanase	0.21 ± 0.00 ^b^	0.21 ± 0.00	0.15 ± 0.04	1.94 ± 1.18	1.53 ± 0.84 ^b^
		Pectinase	0.47 ± 0.13 ^ab^	0.26 ± 0.11	0.21 ± 0.04	3.43 ± 0.35	1.44 ± 0.17 ^b^
	WB_(80–100)_	Xylanase	0.94 ± 0.22 ^ab^	0.24 ± 0.02	0.18 ± 0.26	3.31 ± 0.29	3.83 ± 0.51 ^a^
		Pectinase	1.13 ± 0.53 ^a^	0.25 ± 0.01	0.34 ± 0.08	3.56 ± 0.69	3.73 ± 0.07 ^a^
Residue fraction of F_(200–450)_	WB_(20–40)_	Xylanase	1.56 ± 0.82	2.35 ± 0.43 ^a^	0.04 ± 0.00 ^b^	1.24 ± 0.05 ^b^	13.77 ± 1.72 ^b^
		Pectinase	0.46 ± 0.12	1.21 ± 0.43 ^b^	0.08 ± 0.00 ^ab^	2.83 ± 0.12 ^a^	24.03 ± 0.76 ^a^
	WB_(80–100)_	Xylanase	1.54 ± 0.79	2.26 ± 0.39 ^a^	0.12 ± 0.03 ^a^	1.76 ± 0.09 ^b^	18.36 ± 1.01 ^ab^
		Pectinase	1.42 ± 1.41	1.18 ± 0.16 ^b^	0.12 ± 0.00 ^a^	2.44 ± 0.38 ^a^	20.30 ± 3.96 ^a^
Residue fraction of F_(450+)_	WB_(20–40)_	Xylanase	4.57 ± 1.65 ^b^	3.10 ± 0.02	0.06 ± 0.00 ^a^	1.42 ± 0.13 ^b^	23.22 ± 1.24 ^b^
		Pectinase	4.92 ± 0.05 ^b^	3.34 ± 1.36	0.07 ± 0.00 ^a^	2.18 ± 0.10 ^a^	21.49 ± 2.95 ^b^
	WB_(80–100)_	Xylanase	2.37 ± 1.15 ^b^	3.30 ± 0.43	0.07 ± 0.01 ^a^	0.64 ± 0.18 ^c^	40.29 ± 8.56 ^a^
		Pectinase	3.37 ± 0.23 ^a^	3.96 ± 0.17	0.01 ± 0.01 ^b^	0.65 ± 0.46 ^c^	32.39 ± 5.20 ^ab^

Note: WB_(20–40)_: coarse wheat bran fraction; WB_(80–100)_: the WB fraction with detached AL cell clusters after centrifugal impact milling; F_(−200)_: the fraction retained over the 200-mesh sieve; F_(200–450)_: the fraction retained between the 200- and 450-mesh sieves; F_(450+)_: the fraction passing through the 450-mesh sieve; nd: not detected; different superscript letters denote significant differences among data within the same column (*p* < 0.05).

## Data Availability

The original contributions presented in this study are included in the article. Further inquiries can be directed at the corresponding author.
